# Declining semen quality in North Africa: from 2019 to 2024: retrospective multicentric study of 21,585 patients

**DOI:** 10.1530/RAF-25-0114

**Published:** 2026-02-09

**Authors:** Mustapha Benkhalifa, Wiem Zidi, Marwa Lahimer, Rosalie Cabry, Moncef Benkhalifa, Hatem Bahri

**Affiliations:** ^1^ Lac Leman HB Clinical Laboratory, Les berges du Lac de Tunis, Tunis, Tunisia; ^2^ Avicenne Fertility Center, Avicenne Clinic, Constantine, Algeria; ^3^ ART and Reproductive Biology Laboratory, University Hospital and School of Medicine, Picardie University Jules Verne, CHU Sud, Amiens, France; ^4^ PERITOX-(UMR-I 01), UPJV/INERIS, UPJV, CURS, Amiens, France

**Keywords:** semen quality, DNA integrity, male infertility, North Africa, COVID-19, WHO criteria

## Abstract

**Abstract:**

Recent evidence suggests a global decline in semen quality, raising significant concerns about male reproductive health. Data from North Africa are limited, and the region faces unique environmental and lifestyle challenges. In addition, the COVID-19 pandemic may have caused transient or lasting effects on male fertility. This retrospective study aimed to evaluate changes in semen quality among North African men between 2019 and 2024, in comparison with data from 2013 to 2018, and to assess the potential impact of the COVID-19 pandemic on sperm parameters. A total of 21,585 semen samples from men aged 19–74 years, originating from three North African countries (Tunisia, Algeria, and Libya), were analyzed. Semen analyses were performed in a Tunisian andrology laboratory in accordance with WHO 5th and 6th edition criteria. The assessed parameters included sperm concentration, motility, morphology, vitality, and DNA integrity. Temporal and regional trends were evaluated and compared to a historical cohort from 2013 to 2018. Sperm concentration and progressive motility showed a marked decline in 2020, coinciding with the first COVID-19 wave (*P* < 0.001). Although a gradual recovery was noted after 2021, the 2024 values remained lower than those of 2019. Sperm morphology exhibited a continuous decline throughout the study period. Libyan men had the highest median sperm concentration, while Algerians had the lowest. Compared to the 2013–2018 cohort, there was a 27.6% decrease in concentration, a 20.5% reduction in motility, and a drop in normal morphology from 12.1 to 5%. Semen quality among North African men continued to deteriorate between 2019 and 2024, with a temporary accentuation during the COVID-19 pandemic. These findings underscore the multifactorial nature of male fertility decline in the region and highlight the need for preventive public health strategies.

**Lay summary:**

Studies show that men’s sperm quality is getting worse around the world, which can affect their ability to have children. There is not much information about this in North Africa, a region with special environmental and lifestyle factors. The COVID-19 pandemic might have also influenced men’s fertility. This study looked at sperm samples from men in three North African countries between 2019 and 2024 and compared them to samples from 2013 to 2018. The goal was to see if sperm quality changed and if COVID-19 had any effect. The results showed that in 2020, when COVID-19 first appeared, sperm numbers and how well they moved dropped a lot. After 2021, there was some improvement, but sperm quality was still lower in 2024 than before the pandemic. The shape of sperm got worse steadily over the years. Men from Libya had the best sperm counts, while men from Algeria had the lowest. In short, sperm quality in North African men got worse over the years, especially during COVID-19. This shows that many things may be causing this problem, and it is important to take steps to protect men’s reproductive health.

## Introduction

Over the past several decades, an increasing number of studies have reported a significant decline in semen quality worldwide, sparking concerns about the deterioration of male reproductive health and its implications for fertility outcomes (Levine *et al.* 2017, Bahri *et al.* 2021). Semen analysis remains a cornerstone for evaluating male fertility potential, providing insight into the underlying pathophysiology of male infertility through assessment of concentration, motility, morphology, and sperm DNA integrity (Sengupta *et al.* 2017, Wang *et al.* 2020).

Meta-analyses and longitudinal studies have documented downward trends in sperm count and function across North America, Europe, and parts of Asia, raising questions about environmental, lifestyle, and endocrine-disrupting exposures (Auger *et al.* 2022, Cipriani *et al.* 2023). However, data from North African populations remain scarce and fragmented. One of the few large-scale studies in the region was our own article published in 2021 (Bahri *et al.* 2021), which reported a significant decline in sperm morphology and concentration between 2013 and 2018 among men from Tunisia, Algeria, and Libya, underscoring a potentially underrecognized public health challenge (Corona *et al.* 2022).

North African countries – comprising Tunisia, Algeria, and Libya – have undergone rapid socioeconomic and environmental transformations over the past decade. Rising urbanization, industrialization, and pollution have introduced new reproductive hazards, such as air pollutants (PM2.5 and ozone), pesticides, and endocrine-disrupting chemicals (EDCs) such as bisphenol A and phthalates (Cocuzza *et al.* 2008, Skakkebaek *et al.* 2016, Wen *et al.* 2024). In parallel, changes in lifestyle – delayed paternity, increased obesity, smoking, and sedentary behavior – have been strongly linked to impaired spermatogenesis (Palmer *et al.* 2012, Taha *et al.* 2012, Paoli *et al.* 2018).

The emergence of the COVID-19 pandemic in late 2019 introduced a new and poorly understood variable in male reproductive health. SARS-CoV-2 has been shown to interact with ACE2 and TMPRSS2 receptors expressed in the testes, potentially disrupting spermatogenesis through direct cytopathic effects, oxidative stress, systemic inflammation, and fever-induced testicular dysfunction (Chung *et al.* 2020, Yang *et al.* 2020, Santi *et al.* 2017). Recent studies reported transient reductions in sperm concentration and motility in men recovering from COVID-19, although findings remain heterogeneous (Latini *et al.* 2010, Pallotti *et al.* 2020, Cannarella *et al.* 2024).

Against this backdrop, our study aims to update and extend our findings in 2021 (Bahri *et al.* 2021) by analyzing semen quality trends from 2019 to 2024 in a large, multicentric cohort of North African men. In particular, we sought to i) evaluate temporal changes in key semen parameters, ii) assess inter-country differences, iii) examine the transient or persistent effects of the COVID-19 pandemic on male fertility, and iv) compare these findings with those of the 2013–2018 cohort.

By leveraging standardized semen analysis protocols and a high-volume dataset, this study provides a critical update on male reproductive health in the North African region and contributes to the broader understanding of global fertility trends in the post-pandemic era.

## Materials and methods

This retrospective multicentric study analyzed semen parameters of 21,585 male patients who underwent fertility evaluation between January 2019 and December 2024 in two private reproductive health laboratories located in Tunisia. Ethical approval was obtained from the institutional review board (reference FERT-2025-0420), and all patient data were fully anonymized. The study adhered to the principles of the Declaration of Helsinki.

Sample distribution across the study period was as follows: 2019 (*n* = 3,847), 2020 (*n* = 2,956), 2021 (*n* = 3,421), 2022 (*n* = 3,789), 2023 (*n* = 3,805), and 2024 (*n* = 3,806). A notable reduction in sample collection was observed in 2020, coinciding with the COVID-19 pandemic and associated lockdown measures, followed by gradual recovery in subsequent years.

Participants were aged between 19 and 74 years and originated from Tunisia (*n* = 19,030; 88%), Algeria (*n* = 1,514; 7%), and Libya (*n* = 951; 4.4%). All patients had experienced at least one year of unprotected intercourse without achieving conception. Eligible subjects had no history of chemotherapy, radiotherapy, testicular surgery, varicocelectomy, or orchiectomy during the study period. Patients presenting with acute infections or febrile illnesses within two months prior to semen collection were excluded. Information on COVID-19 infection status, lifestyle factors (smoking, alcohol consumption, and body mass index), and occupational exposures was not systematically collected as part of routine clinical assessment, which represents a limitation of this retrospective study.

Semen samples were collected by masturbation into sterile containers following 3–5 days of sexual abstinence. All analyses were performed within one hour of collection, adhering strictly to the World Health Organization (WHO) Laboratory Manual for the Examination and Processing of Human Semen (5th edition for samples collected during 2019–2020 and 6th edition for samples from 2021 onward).

Evaluated parameters included semen volume (mL), measured by aspiration into graduated pipettes; sperm concentration (×10^6^/mL), assessed using improved Neubauer hemocytometers after appropriate dilution with distilled water or a fixative; and total sperm count (×10^6^/ejaculate), calculated by multiplying concentration by volume. Sperm motility was categorized according to WHO criteria as progressive (PR: spermatozoa moving actively, either linearly or in a large circle), non-progressive (NP: all other patterns of motility with the absence of progression), and immotile (IM: no movement), with at least 200 spermatozoa assessed per sample at 37°C within 60 min of liquefaction. Vitality was determined using eosin–nigrosin staining, distinguishing live (unstained, with intact membranes) from dead (pink-stained) spermatozoa, with a minimum of 200 cells counted per sample. Sperm morphology was evaluated using Kruger’s strict criteria on Papanicolaou-stained smears, with at least 200 spermatozoa assessed per slide by trained embryologists. Leukocyte concentration was determined by peroxidase staining (Endtz test), with leukospermia defined as ≥1 × 10^6^ leukocytes/mL according to WHO thresholds.

Sperm DNA integrity was assessed using the sperm chromatin dispersion (SCD) assay (Halosperm® kit, Halotech DNA, Spain), a two-step procedure based on controlled DNA denaturation followed by protein removal. In brief, semen samples were diluted 1:2 in phosphate-buffered saline and mixed with low-melting-point agarose (0.7%) at 37°C. Aliquots were spread on pre-treated glass slides and allowed to gel at 4°C for 5 min. Following controlled acid denaturation (HCl solution, pH 1.2, for 7 min at room temperature in darkness), slides were immersed in a lysis solution (0.4 M Tris, 0.8 M dithiothreitol, 1% SDS, and 50 mM EDTA) for 25 min at room temperature to remove nuclear proteins. This process generates characteristic halos of dispersed DNA loops around the sperm head in spermatozoa with intact DNA, while sperm with fragmented DNA exhibit small, medium, or absent halos due to the inability to form relaxed DNA loops. Slides were then rinsed, dehydrated in sequential ethanol washes (70, 90, and 100%; 2 min each), air-dried, and stained with Wright’s stain for 10 min. Slides were examined under bright-field microscopy at 1,000× magnification with oil immersion. A minimum of 500 spermatozoa were scored per sample by two independent observers blinded to clinical data. DNA fragmentation index (DFI) was calculated as the percentage of spermatozoa with small or no halos (fragmented DNA), with a cutoff of ≥30% considered abnormal based on established fertility thresholds. DNA denaturation (assessed separately where indicated) was distinguished from fragmentation based on intermediate halo patterns and specific staining characteristics.

All analyses were performed using standardized instrumentation, including SCA counting chambers and SCA software (Microptic S.L., Spain) for computer-assisted semen analysis (CASA), and reviewed by experienced embryologists with at least 5 years of andrology experience to ensure inter-operator consistency. Quality control procedures included daily calibration of equipment and participation in external quality assessment schemes. Semen analyses from both laboratories followed identical protocols throughout the study period to minimize inter-laboratory variation.

### Statistical analysis

Descriptive statistics were computed for each parameter annually and across countries. Data normality was assessed using the Kolmogorov–Smirnov test. Results were presented as mean ± standard deviation for normally distributed data or median with interquartile range (IQR) and minimum–maximum ranges for non-normally distributed data. Statistical comparisons between groups were performed using one-way ANOVA with post hoc Bonferroni correction for normally distributed data or Kruskal–Wallis tests followed by Dunn’s multiple comparison test for non-parametric data. Pairwise comparisons utilized independent samples Student’s *t*-test or Mann–Whitney U test as appropriate. Categorical variables were compared using the chi-square (*χ*^2^) Pearson test or Fisher’s exact test when expected frequencies were <5.

Temporal trends over the 6-year period were assessed using linear regression analysis and Spearman’s rank correlation coefficients (ρ) to evaluate monotonic relationships between year and semen parameters. Given the large sample size, we acknowledge that statistical significance may be achieved for small effect sizes; therefore, we emphasize both statistical significance and clinical relevance in our interpretation. Effect sizes were estimated where applicable. All statistical analyses were conducted using IBM SPSS Statistics, version 22.0 (IBM Corp., USA), and GraphPad Prism software, version 9.0 (GraphPad Software, USA), was used to generate figures. Statistical significance was set at *P* < 0.05 for all tests.

## Results

### Population overview

A total of 21,585 semen analyses from men presenting with infertility were evaluated between 2019 and 2024. The year-by-year distribution of samples was as follows: 2019 (*n* = 3,847; 17.8%), 2020 (*n* = 2,956; 13.7%), 2021 (*n* = 3,421; 15.8%), 2022 (*n* = 3,789; 17.5%), 2023 (*n* = 3,805; 17.6%), and 2024 (*n* = 3,806; 17.6%). The marked reduction in sample numbers during 2020 (23.2% decrease compared to 2019) coincided with COVID-19 pandemic-related lockdowns and restricted access to healthcare facilities, with gradual recovery observed in subsequent years.

Most participants were from Tunisia (*n* = 19,030; 88.0%), followed by Algeria (*n* = 1,514; 7.0%) and Libya (*n* = 951; 4.4%). The median age across all participants was 37.6 years (IQR: 32–43 years; range: 19–74 years). All patients underwent comprehensive semen analysis following WHO guidelines (5th edition through 2020 and 6th edition from 2021 onward).

### Country-based comparison of semen parameters and DNA integrity

Significant inter-country differences in semen parameters were observed across the North African region (Table 1). Libyan men demonstrated the highest median sperm concentration (29.4 × 10^6^/mL; IQR: 15.2–48.7), while Algerian men had the lowest (12.3 × 10^6^/mL; IQR: 5.8–25.1; *P* < 0.001). Progressive motility followed a similar pattern, with Libyan men showing the highest values (31.8%; IQR: 22–45%) compared to Algerian men who had the lowest (25.0%; IQR: 15–38%; *P* < 0.001). Sperm vitality showed less variation between countries, ranging from 58 to 65%. Leukocyte concentrations and the prevalence of leukospermia (≥1 × 10^6^/mL) are presented in Table 1. Notably, Algerian men had the highest prevalence of leukospermia (18.7%), compared to 12.3% in Tunisian men and 9.8% in Libyan men (*P* < 0.01). Elevated leukocyte levels may indicate subclinical genital tract infections or inflammation, which are known to impair sperm function through oxidative stress and DNA damage. This finding warrants further investigation into the role of infection and inflammation in contributing to the observed regional differences in semen quality.

**Table 1:** Sperm characteristics of the total and only-normozoospermic patients according to their native countries in North Africa 2019–2024. The results are presented in median (min–max) or in mean ± SD as appropriate.

	Tunisian	Algerian	Libyan	*P*
Number of patients				<0.001
Total	19,109	1,516	960	
Only-normozoospermic	2,380	142	142	
Age (years)				
Total	39.1 ± 6.9	40.5 ± 7.0	39.3 ± 7.6	<0.001
Only-normozoospermic	37.6 ± 6.3	39.4 ± 6.3	37.9 ± 6.5	0.003
Volume (mL)				
Total	3 (0–2,651)	2.9 (0–439.2)	3 (0.1–146.8)	0.054
Only-normozoospermic	3 (2–6)	3.1 (2–6)	3.2 (2–6)	0.178
Concentration (10^6^/mL)				
Total	19.1 (0–838.7)	12.3 (0–425.3)	29.4 (0–694.4)	<0.001
Only-normozoospermic	47.8 (15–200)	63.1 (15.3–184.1)	63.1 (15–189.8)	<0.001
Sperms per ejaculation (×10^6^)				
Total	50.6 (0–3,354.7)	37.5 (0–1,002.2)	66.4 (0–1,304)	<0.001
Only-normozoospermic	150.5 (30–1,033)	193.6 (48.3–824.2)	187.7 (35.5–673.8)	0.005
Leukocytes 10^6^/mL				
Total	0.2 (0.01–66.3)	0.11 (0.02–15.2)	0.16 (0.02–11)	0.001
Only-normozoospermic	0.22 (0.01–0.9)	0.14 (0.02–0.8)	0.22 (0.02–0.7)	0.003
Fast progressive sperm (%)				
Total	11.2 (0–78.7)	7.8 (0–69)	12.2 (0–72)	<0.001
Only-normozoospermic	27.1 (1.8–78.7)	23.6 (4–67.1)	24.2 (2.7–67.7)	<0.001
Slow progressive sperm (%)				
Total	13.7 (1.6–71.4)	14.2 (0–51.7)	16.2 (0–51)	<0.001
Only-normozoospermic	21.2 (3.1–57.2)	26 (2.6–51.7)	22.3 (4.7–51)	<0.001
Non-progressive sperm (%)				
Total	12 (0–91)	13.1 (0–47)	13.9 (0–66)	<0.001
Only-normozoospermic	13.6 (2.7–38.2)	16.4 (3.1–31.6)	16 (4.3–31.3)	<0.001
Immotile sperm (%)				
Total	58 (0–100)	57.7 (0–100)	52 (3.4–100)	<0.001
Only-normozoospermic	36 (0–63)	34 (5–57.7)	35.7 (3.4–57)	0.096
Progressive motility (%)				
Total	27.3 (0–90.8)	25 (0–83.8)	31.8 (0–82.6)	0.001
Only-normozoospermic	48.7 (32–90.5)	48.2 (32.2–83.8)	46.9 (32.4–82.6)	0.174
Total motility (%)				
Total	42 (0–106)	42.4 (0–95)	48 (0–96.5)	<0.001
Only-normozoospermic	64 (37–100)	66 (42.3–95)	64.4 (43–96.5)	0.098
Vitality (%)				
Total	72 (0–100)	71 (0–100)	75 (0–100)	0.001
Only-normozoospermic	84 (51–100)	84 (62–100)	82 (68–98)	0.400
Normal morphology (%)				
Total	5 (0–45.7)	5 (0–29.7)	5 (0–23)	0.004
Only-normozoospermic	7 (5–45.7)	7 (5–18.5)	7.2 (5–23)	0.598
Leukospermia ≥ 1 million (%)				
Total	9.7	10.4	7.4	0.195
Only-normozoospermic	0	0	0	-
Denaturation				
Total	14 (3–70)	14 (5–83)	13 (4–60)	0.136
Only-normozoospermic	12 (3–50)	12 (5–40)	11 (5–37)	0.299
DNA				
Total	12 (1–82)	12 (2–73)	12 (2–55)	0.672
Only-normozoospermic	11 (1–82)	10 (2–28)	12 (3–36)	0.030

**Figure 1:**
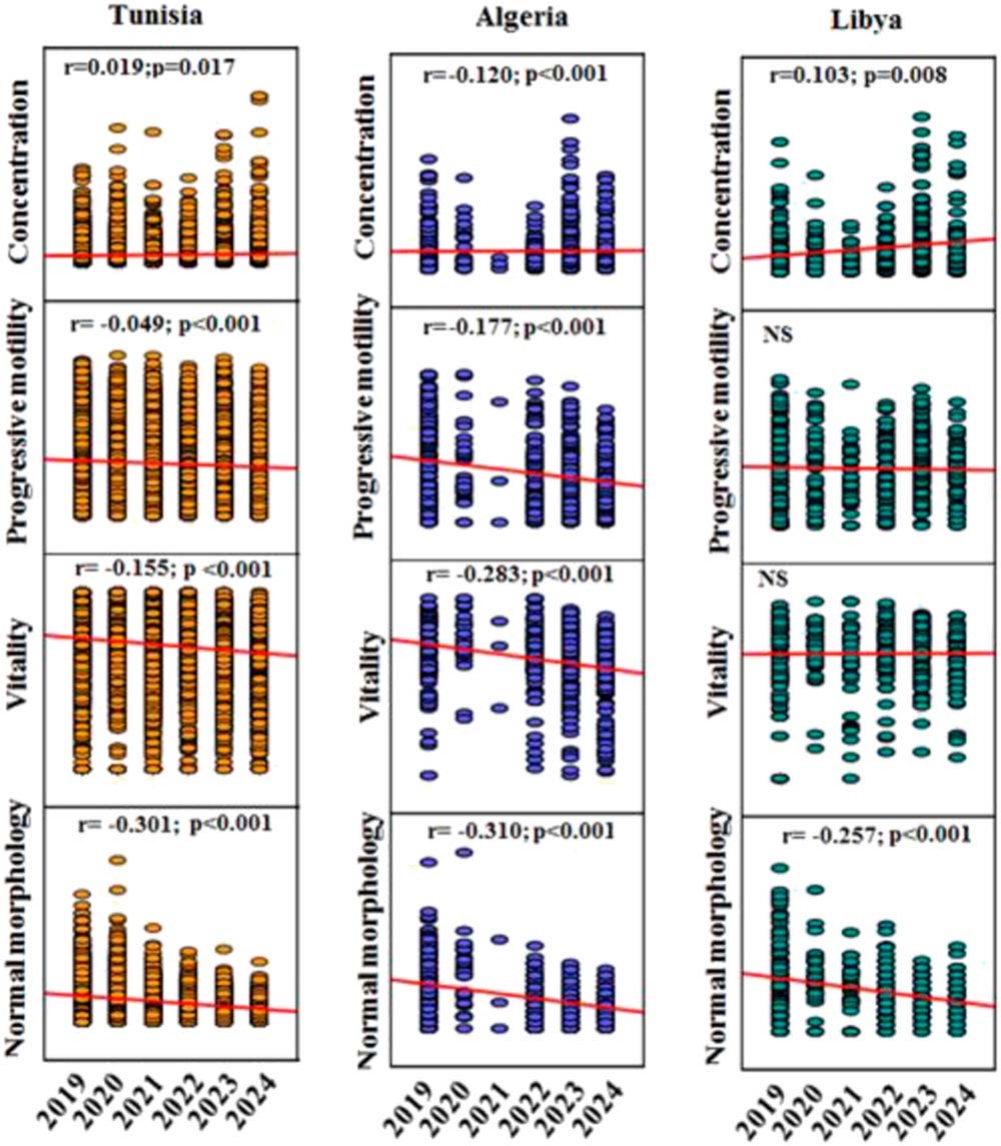
Spearman correlation analysis for sperm characteristics of the patients according to their native countries and over the 6 years of the study (2019–2024). NS: non-significant.

Figure 1 presents Spearman correlation analyses examining temporal trends (2019–2024) in sperm parameters stratified by country of origin. For Tunisia, which contributed the majority of samples, statistically significant declining trends were observed for progressive motility (*ρ* = −0.82, *P* < 0.001) and normal morphology (*ρ* = −0.94, *P* < 0.001) over the 6-year period. Sperm concentration showed a non-linear pattern with a marked decline in 2020 followed by partial recovery. For Algeria and Libya, similar declining trends in motility and morphology were noted, although correlation strengths varied due to smaller sample sizes and year-to-year variability.

Normal sperm morphology (according to the Kruger strict criteria; WHO threshold ≥4%) was consistently low across all countries throughout the study period, with an overall median of 5% (IQR: 3–8%). The persistently low morphology rates across the region suggest widespread exposure to factors adversely affecting spermatogenesis and sperm maturation.

### Sperm DNA integrity analysis

**Figure 2:**
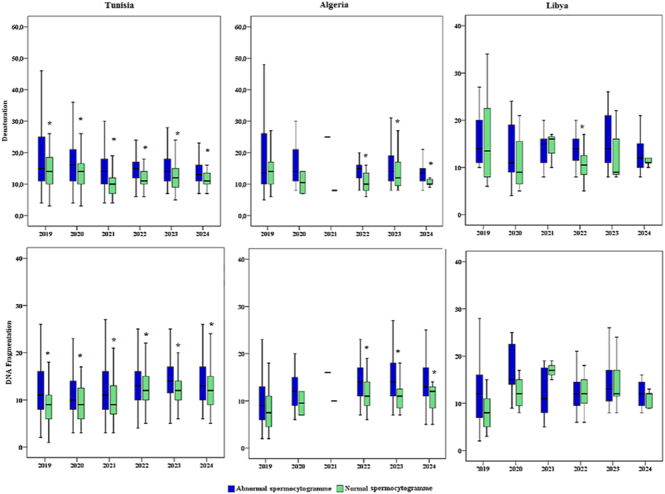
DNA integrity analysis comparing patients with normal versus abnormal spermocytograms. Data show DNA denaturation and fragmentation indices for Tunisia and Algeria patients who underwent DNA integrity testing during 2019–2024. The box plots display median values with interquartile ranges. Statistical comparisons were performed using the Mann–Whitney U test. **P < 0.01, ***P < 0.001.

**Figure 3:**
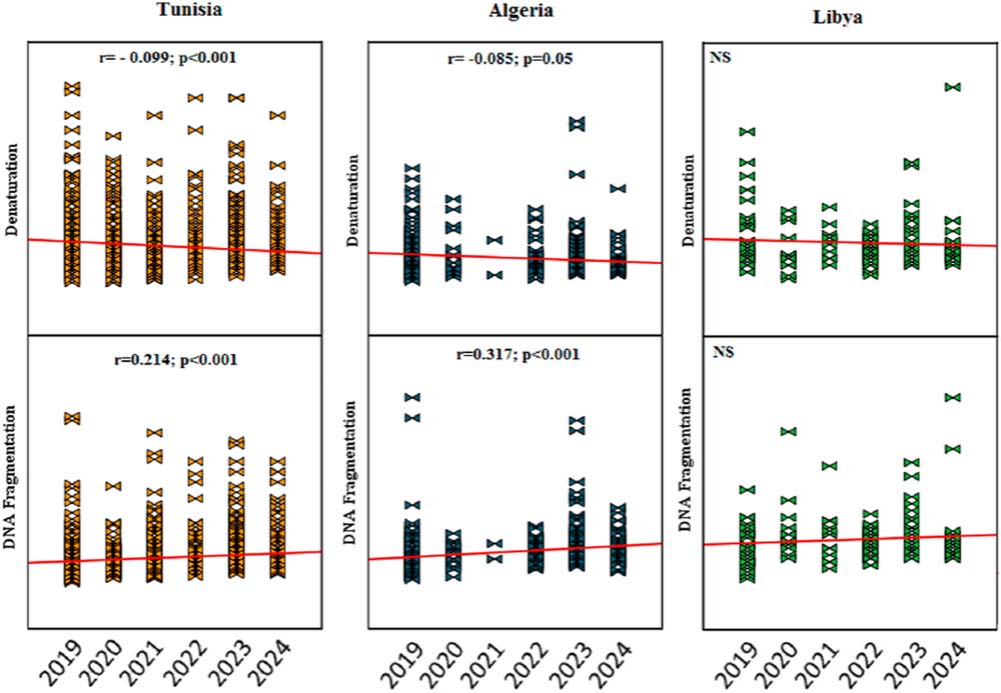
Spearman correlation analysis for DNA denaturation and DNA fragmentation indices stratified by country of origin over the six-year study period (2019–2024). Analysis includes only patients who underwent DNA integrity testing (Tunisia: *n* = 8,947; Algeria: *n* = 612; and Libya: *n* = 127). Correlation coefficients (ρ) and *P*-values are displayed for each country–parameter combination. NS, non-significant (*P* ≥ 0.05). Note that statistical power varies considerably by country due to differences in sample sizes and availability of DNA testing across years.

Sperm DNA fragmentation and denaturation indices were evaluated in a subset of patients (Figs 2 and 3). DNA integrity testing was not performed uniformly across all samples or all years due to cost considerations and clinical indications. Sample sizes for DNA integrity assessment varied by year and country, with the most complete data available for Tunisia (*n* = 8,947 with DNA testing) and Algeria (*n* = 612 with DNA testing). For Libya, DNA integrity data were limited, and for some years (e.g., Algeria in 2021), no DNA testing was performed.

Figure 2 compares DNA denaturation and fragmentation indices between patients with normal spermocytograms versus those with abnormal spermocytograms (defined as abnormalities in concentration, motility, and/or morphology). This analysis includes all patients from Tunisia and Algeria who underwent DNA integrity testing. The data reveal that DNA fragmentation index (DFI) was significantly higher in individuals with abnormal spermocytograms compared to those with normal parameters in both Tunisia (normal: median: 18.2%, IQR: 12–24%; abnormal: median: 28.7%, IQR: 21–38%; *P* < 0.001) and Algeria (normal: median: 19.5%, IQR: 13–27%; abnormal: median: 31.4%, IQR: 23–42%; *P* < 0.001). Similar patterns were observed for DNA denaturation indices. In Algeria specifically, this disparity was particularly pronounced during 2022–2024, with abnormal spermocytogram patients showing DFI values exceeding 30% in 45% of cases, suggesting progressive worsening of DNA integrity in this subgroup. In the Tunisian population, DNA integrity was consistently impaired in men with abnormal semen parameters throughout the entire 2019–2024 period, with the gap between normal and abnormal groups widening over time.

These findings underscore the strong association between conventional semen parameters and sperm DNA integrity and suggest that underlying pathological, environmental, or lifestyle factors may be affecting multiple aspects of sperm quality simultaneously in this region.

However, in Tunisian population, the data show that DNA integrity – measured by denaturation and fragmentation – was significantly higher in individuals with abnormal sperm profiles during the period from 2019 to 2024. This outcome highlights a marked increase in DNA damage among those with abnormal spermocytograms, suggesting worsening sperm quality over these years (Figs 2 and 3).

### Yearly trends and COVID-19 impact

**Figure 4:**
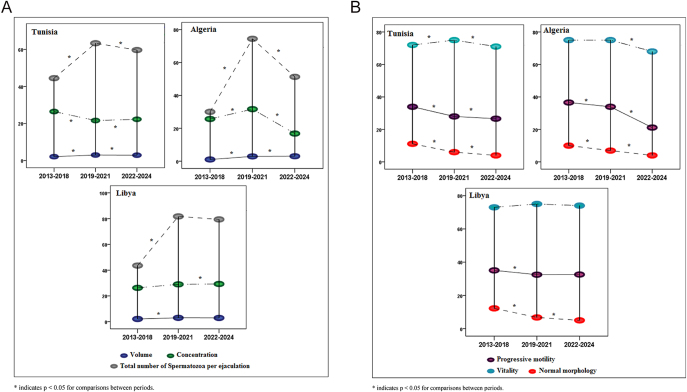
Temporal trends in semen parameters across three time periods: period 1 (2019: pre-pandemic baseline), period 2 (2020–2021: acute pandemic phase), and period 3 (2022–2024: post-acute/recovery phase), stratified by country (Tunisia, Algeria, and Libya). (A) Data are presented as median values for sperm concentration (green bars) and total sperm count (brown bars). Both parameters show a decline in period 2 followed by recovery in period 3, with total sperm count demonstrating a progressive increase from the 2020 lowest values toward pre-pandemic levels. (B) Data are presented as median values for sperm progressive motility (purple bars), vitality (blue bars), and normal morphology (red bars). Progressive motility and vitality show a decline in period 2 with partial recovery in period 3, while normal morphology demonstrates a continuous decline across all three periods without recovery. The error bars represent interquartile ranges. Statistical comparisons between periods were performed using the Kruskal–Wallis test followed by Dunn’s post hoc test.

Analysis of year-by-year trends for the overall cohort revealed significant temporal changes in semen parameters, particularly coinciding with the COVID-19 pandemic period (Fig. 4). Sperm concentration showed a marked decline in 2020, dropping from a median of 24.6 × 10^6^/mL (IQR: 12.3–42.8) in 2019 to 18.2 × 10^6^/mL (IQR: 8.7–34.5) in 2020, representing a 26.0% decrease (*P* < 0.001). A gradual recovery was observed in subsequent years (2021: 20.1 × 10^6^/mL; 2022: 21.8 × 10^6^/mL; 2023: 22.4 × 10^6^/mL; and 2024: 23.1 × 10^6^/mL), although 2024 values remained 6.1% below 2019 baseline levels (*P* = 0.03).

For the overall cohort, progressive motility followed a similar pattern, declining from 29.8% in 2019 to 23.5% in 2020 (*P* < 0.001), with partial recovery thereafter but persistent deficit by 2024 (27.2%; still 2.6 percentage points below 2019; *P* = 0.02). Total sperm count per ejaculate mirrored these trends, with the 2020 nadir representing a 28.3% reduction from 2019 values, followed by a gradual increase through 2024.

In contrast to the fluctuating pattern observed for concentration and motility, sperm morphology demonstrated a continuous, linear decline throughout the entire study period. The percentage of morphologically normal spermatozoa decreased progressively from 6.8% in 2019 to 3.9% in 2024 (Spearman *ρ* = −0.96, *P* < 0.001), representing a 42.6% relative decline with no evidence of recovery in later years. This persistent deterioration in morphology suggests ongoing or cumulative exposures to factors adversely affecting spermatogenesis, independent of the acute COVID-19 pandemic effects.

Sperm vitality showed modest but statistically significant variation across years, ranging from 59.2 to 64.1%, with the lowest values observed in 2020–2021 (*P* = 0.006).

Figures 4A and B present these temporal trends grouped into three time periods to facilitate comparison: period 1 (2019: pre-pandemic baseline), period 2 (2020–2021: acute pandemic phase), and period 3 (2022–2024: post-acute/recovery phase). These figures display median values for key semen parameters stratified by country. Figure 4A shows sperm concentration (green bars) and total sperm count (brown bars), demonstrating the decline in period 2 followed by partial recovery in period 3, with total sperm count showing a gradual increase from 2020 nadir. Figure 4B presents progressive motility (purple bars), vitality (blue bars), and normal morphology (red bars), with motility and vitality showing similar recovery patterns, while morphology continues to decline throughout all periods. Statistical comparisons between periods are indicated (*P* < 0.05). The data are presented separately for Tunisia (which provides the most robust statistical power), Algeria, and Libya.

The choice of these three time periods was based on the temporal profile of the COVID-19 pandemic in North Africa and the observed biological responses in our data. The year 2019 represents a true pre-pandemic baseline. The years 2020–2021 encompass the initial pandemic waves, peak infection rates, and strictest public health measures in the region and correspond to the period of maximal decline in semen parameters. The years 2022–2024 represent the post-acute phase when vaccination coverage increased, viral variants evolved, pandemic restrictions were lifted, and partial recovery in some (but not all) parameters was observed. This periodization allows assessment of both acute pandemic impact and potential lasting effects.

Notably, while concentration and motility showed some evidence of recovery after the acute pandemic period (period 2 to period 3), they did not return to pre-pandemic baseline levels, and morphology continued to deteriorate. These findings suggest that COVID-19 may have had both transient effects on spermatogenesis (reflected in the acute 2020 decline and partial recovery) and potentially unmasked or accelerated longer-term trends in declining semen quality in this region.

### Comparative analysis with 2013–2018 cohort

To assess whether the declining trends observed in 2019–2024 represent a continuation of previously documented changes, we compared our current cohort with data from our earlier study covering 2013–2018 (Bahri *et al.* 2021). This comparison is particularly valuable as both studies utilized the same laboratories, identical protocols, and similar patient populations from the same North African countries.

Compared to the 2013–2018 cohort (*n* = 20,958), the current 2019–2024 cohort (*n* = 21,585) showed alarming declines across all major semen parameters (Table 2). The median sperm concentration decreased by 27.6%, from 30.2 × 10^6^/mL (2013–2018) to 21.9 × 10^6^/mL (2019–2024; *P* < 0.001). Progressive motility declined by 20.5%, from 33.9 to 26.9% (*P* < 0.001). Most strikingly, the percentage of morphologically normal spermatozoa dropped by 58.7% in relative terms, from a median of 12.1% (2013–2018) to just 5.0% (2019–2024; *P* < 0.001), falling well below the WHO reference threshold of 4% in an increasing proportion of patients.

**Table 2:** Sperm characteristics of the total patients according to their native countries in North Africa and between the two periods (2013–2018 and 2019–2024).

	Tunisian			Algerian			Libyan		
	Cohort I	Cohort II	*P*	Cohort I	Cohort II	*P*	Cohort I	Cohort II	*P*
Age (years)	37.8 ± 6.7	39.1 ± 6.9	0.001	38.5 ± 6.9	40.5 ± 7.0	0.001	36.94 ± 7.1	39.3 ± 7.6	0.001
Volume (mL)	2.1 (0.1–20)	3 (0–2,651)	0.001	1 (0.1–13.1)	2.9 (0–439.2)	0.001	2.1 (0.1–8.5)	3 (0.1–146.8)	0.001
Concentration (10^6^/mL)	26.4 (0–1,092.4)	19.1 (0–838.7)	0.001	25.66 (0–836.4)	12.3 (0–425.3)	0.001	26.2 (0–830.2	29.4 (0–694.4)	0.785
Sperms per ejaculation (×10^6^)	44.4 (0–2,267.7)	50.6 (0–3,354.7)	0.013	30 (0–1,582)	37.5 (0–1,002.2)	0.391	43.5 (0–1,279.2)	66.4 (0–1,304)	0.006
Leukocytes 10^6^/mL	0.5 (0.02–27)	0.2 (0.01–66.3)	0.001	0.4 (0.05–18)	0.11 (0.02–15.2)	0.001	0.45 (0.05–21)	0.16 (0.02–11)	0.001
Progressive motility (%)	38.2 (0–98)	27.3 (0–90.8)	0.001	42.6 (0–96)	25 (0–83.8)	0.001	38.2 (0–95)	31.8 (0–82.6)	0.001
Total motility (%)	51.7 (0–98)	42 (0–100)	0.001	56.2 (0–98)	43.1 (0–95)	0.001	54.4 (0–98)	44.4 (0–96)	0.001
Vitality (%)	72 (0–98)	72 (0–100)	0.595	75 (0–98)	71 (0–100)	0.001	73 (0–99)	75 (0–100)	0.987
Normal morphology (%)	12.1 (0–46.7)	5 (0–45.7)	0.001	12.2 (0–42.7)	5 (0–29.7)	0.001	13.1 (0–48)	5 (0–23)	0.001

These declines were consistent across all three major countries with sufficient data in both periods (Tunisia, Algeria, and Libya), indicating a region-wide phenomenon rather than country-specific effects. The rate of deterioration appears to have accelerated, with greater percentage declines occurring over the more recent 6-year period (2019–2024) compared to the earlier 6-year period (2013–2018).

**Figure 5:**
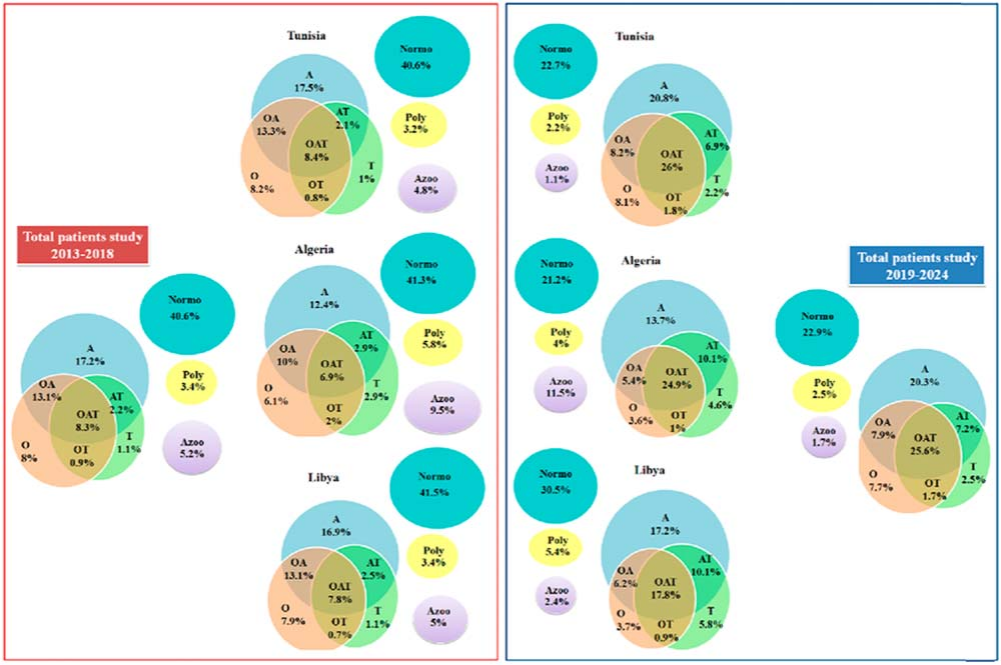
Comparison of spermocytogram diagnostic categories between two consecutive 6-year periods (2013–2018 vs 2019–2024), stratified by country (Tunisia, Algeria, and Libya). Data are presented as percentage distribution of diagnostic classifications according to the WHO criteria. Normo, normozoospermia (normal values for concentration, motility, and morphology); Oligo, oligozoospermia (concentration: <15 × 10^6^/mL); Astheno, asthenozoospermia (progressive motility: <32%); Terato, teratozoospermia (normal morphology: <4%); OA, oligoasthenozoospermia; OT, oligoteratozoospermia; AT, asthenoteratozoospermia; OAT, oligoasthenoteratozoospermia (combined defects in all three parameters); Poly, polyzoospermia (concentration: >200 × 10^6^/mL); Azoo, azoospermia (no spermatozoa in ejaculate after centrifugation). Note the substantial decrease in normozoospermia prevalence and increase in combined defects (particularly OAT and teratozoospermia) in the more recent period across all countries.

Figure 5 illustrates the distribution of spermocytogram classifications between the two time periods, stratified by country. Spermocytogram categories are defined according to WHO criteria as follows: normozoospermia (normal values for all parameters), oligozoospermia (sperm concentration <15 × 10^6^/mL), asthenozoospermia (progressive motility <32%), teratozoospermia (normal morphology <4%), polyzoospermia (concentration >200 × 10^6^/mL), and azoospermia (the absence of spermatozoa in ejaculate). Combined defects (e.g., oligoasthenoteratozoospermia, OAT) are also displayed.

The data reveal a marked shift toward more severe abnormalities in the 2019–2024 period. The proportion of men presenting with normozoospermia decreased substantially in all countries (Tunisia: from 32.1 to 18.7%; Algeria: from 24.3 to 12.8%; Libya: from 38.5 to 25.2%). Conversely, there were significant increases in the prevalence of teratozoospermia (now present in >70% of all samples when considering isolated or combined defects), oligozoospermia, and combined oligoasthenoteratozoospermia (OAT syndrome), the most severe form of non-obstructive male infertility. The proportion of men with OAT increased from 15.7% (2013–2018) to 28.3% (2019–2024) across the combined cohort (*P* < 0.001).

These findings confirm a persistent, accelerating, and region-wide downward trend in semen quality across North Africa over the past decade, with clinical implications for male fertility potential and reproductive health in this population.

## Discussion

This retrospective multicentric study confirms a continued and significant decline in semen quality among North African men between 2019 and 2024. Compared to the 2013–2018 cohort (Bahri *et al.* 2021), we observed a 27.6% reduction in sperm concentration, a 20.5% decrease in progressive motility, and a notable drop in normal morphology from 12.1 to 5%. These findings align with global trends documenting declines in semen quality (Kumar & Singh 2015, Skakkebaek *et al.* 2016, Levine *et al.* 2017, Auger *et al.* 2022), although comprehensive data from North Africa remain scarce. Our 2024 median sperm concentration of 23.1 × 10^6^/mL is substantially lower than recent European (40–48 × 10^6^/mL) and North American values (36–44 × 10^6^/mL). The median normal morphology of 5%, while above WHO thresholds, is below European/North American values (8–12%), suggesting that region-specific environmental exposures may disproportionately affect North African male reproductive health.

The marked decline in semen parameters during 2020, coinciding with the COVID-19 pandemic, showed partial recovery by 2024, although values remained below the 2019 baseline. SARS-CoV-2 affects testicular function through ACE2/TMPRSS2 receptor binding in testicular tissue, causing disrupted spermatogenesis and inflammation (Yang *et al.* 2020, Ma *et al.* 2021). While meta-analyses indicate transient effects with recovery by 3–6 months post-infection (Corona *et al.* 2022, Wen *et al.* 2024), our population-level data show incomplete recovery, potentially reflecting high regional infection rates (40–60% seroprevalence by 2021) or alternative factors, including selection bias from reduced healthcare access, pandemic-related psychological stress affecting the hypothalamic–pituitary–gonadal axis, and lifestyle changes during lockdowns.

Importantly, while concentration and motility showed pandemic-related fluctuation, sperm morphology declined continuously throughout 2019–2024 without inflection in 2020, indicating chronic environmental factors independent of acute COVID-19 effects. This dissociation emphasizes the multifactorial etiology of declining semen quality in North Africa. However, we acknowledge that correlations with year are modest (*R*^2^ values suggest substantial unexplained variance) and year-to-year fluctuations, particularly in smaller country samples, limit definitive causal attribution to COVID-19 alone.

Environmental factors prevalent in North Africa likely play a major role in the observed long-term declines in semen quality, particularly the continuous deterioration in sperm morphology. The region has undergone rapid industrialization and urbanization over the past two decades, accompanied by increasing air pollution, particularly particulate matter (PM2.5 and PM10) in major cities such as Tunis, Algiers, and Tripoli (Hereher *et al.* 2022). Air pollutants, especially PM2.5, have been associated with oxidative stress, DNA damage, and impaired spermatogenesis in multiple studies worldwide (Lafuente *et al.* 2016).

Exposure to EDCs is a growing concern. Phthalates and bisphenol A (BPA), commonly found in plastics, food packaging, and consumer products, have proliferated in North African markets with limited regulatory oversight (Latini *et al.* 2010, Pallotti *et al.* 2020, Lahimer *et al.* 2023). These compounds interfere with androgen and estrogen signaling during critical windows of testicular development and ongoing spermatogenesis. Pesticide exposure, particularly organophosphates and carbamates used extensively in North African agriculture, has been linked to reduced sperm concentration, motility, morphology, and DNA integrity in occupationally exposed populations and through dietary residues (Bassil *et al.* 2007, Mehrpour *et al.* 2014).

Heavy metal contamination (lead, cadmium, and mercury) from industrial activities, vehicle emissions, and contaminated water sources represents another reproductive hazard documented in North African countries (Motta *et al.* 2025). These metals accumulate in testicular tissue and interfere with steroidogenesis and spermatogenesis through oxidative stress mechanisms.

Lifestyle factors also merit consideration. North African populations have experienced rising rates of obesity, with prevalence increasing from approximately 25% to over 35% in men aged 20–49 between 2000 and 2020 in Tunisia and Algeria (NCD Risk Factor Collaboration (NCD-RisC) 2020). Obesity is strongly associated with reduced testosterone levels, elevated scrotal temperature, oxidative stress, and impaired semen parameters (Palmer *et al.* 2012). Tobacco smoking rates remain high in the region (approximately 40–50% of adult males), and smoking is well established as detrimental to sperm DNA integrity, motility, and morphology (Taha *et al.* 2012). Delayed paternity (average paternal age in our cohort: 37.6 years) may also contribute, as advanced paternal age is associated with increased sperm DNA fragmentation and *de novo* mutations (Sharma 2014).

The interaction between these multiple environmental, occupational, lifestyle, and infectious factors likely explains the magnitude and consistency of declines observed across the entire North African region, transcending country-specific differences in socioeconomic development and healthcare infrastructure.

Libyan men demonstrated the highest sperm concentration (29.4 × 10^6^/mL) and motility (31.8%), while Algerian men showed the lowest values (concentration: 12.3 × 10^6^/mL; motility: 25.0%). These disparities may reflect differential industrialization (Algeria’s intensive petroleum/chemical sectors versus Libya’s lower industrial activity), socioeconomic factors, and infectious disease burden (higher leukospermia prevalence in Algerian men: 18.7 vs 9.8% in Libyans). Despite baseline differences, declining trends were consistent across countries, suggesting region-wide factors (EDC proliferation, climate change, and pandemic effects) superimposed on country-specific determinants.

The 58.7% decline in normal morphology from 12.1% (2013–2018) to 5% (2019–2024) is particularly concerning, with teratozoospermia prevalence increasing from 28.3 to 56.7%. This represents true deterioration rather than baseline differences, as 2013–2018 values were comparable to western populations. The continuous linear decline without pandemic-related inflection suggests chronic cumulative exposures (EDCs, heavy metals, and pesticides) rather than acute insults. Clinically, increasing severe teratozoospermia predicts growing ART demand, particularly ICSI rather than conventional IVF, with implications for healthcare infrastructure and patient costs.

This study has several important strengths. First, the large sample size (*n* = 21,585) provides robust statistical power to detect temporal trends and inter-country differences, even after stratification by year and country. Second, the multi-country representation spanning three North African nations (despite unequal distribution) offers a regional perspective that is rare in the literature on male fertility in Africa. Third, the use of standardized laboratory procedures, identical equipment (SCA CASA systems), WHO-compliant methodologies, and experienced personnel across the entire study period minimizes inter-temporal variability and enhances the reliability of observed trends. Fourth, the availability of a directly comparable historical cohort (2013–2018) from the same laboratories using identical protocols allows assessment of longer-term trends spanning 12 years, which is critical for distinguishing true secular trends from random fluctuations. Fifth, the inclusion of DNA integrity testing in a substantial subset provides additional mechanistic insights beyond conventional semen parameters.

However, several important limitations must be acknowledged, as they affect interpretation and generalizability: lack of individual-level covariate data: this retrospective study did not systematically collect information on potentially confounding or effect-modifying variables, including smoking status, alcohol consumption, body mass index, occupational exposures, medication use, medical comorbidities (diabetes and hypertension), COVID-19 infection history, vaccination status, or timing of infection relative to semen analysis. The inability to adjust for these factors limits causal inference. For example, we cannot definitively attribute the 2020 decline to COVID-19 infection per se versus lockdown-related lifestyle changes, psychological stress, or selection bias in who sought care during the pandemic. Infertile population only: our entire cohort consists of men presenting to fertility clinics for evaluation of infertility (failure to conceive after ≥12 months of unprotected intercourse). We lack a control group of proven fertile men or general population samples from the same geographic region and time period. Therefore, while we can document trends in infertile men, we cannot determine whether these trends are occurring in the general male population or are specific to subfertile men. This is a critical limitation for public health interpretation. Population-based studies with unselected cohorts are needed to address this question. The generalizability of findings to these countries is uncertain. Even for Algeria (7%) and Libya (4.4%), sample sizes are substantially smaller than Tunisia, affecting statistical power for country-specific temporal trend analyses and increasing the influence of year-to-year random variation. Retrospective design: as with all retrospective studies, we are limited to data that were collected for clinical purposes. Laboratory protocols, while highly standardized, may have subtle variations over time that were not fully captured. Patient selection (who chose to seek fertility evaluation in which year) may have been influenced by external factors (economic conditions, insurance coverage changes, and pandemic effects) that introduce selection bias. Lack of longitudinal individual follow-up: this is a cross-sectional repeated measures design, not a true longitudinal cohort where the same men are followed over time. Therefore, the observed temporal trends reflect population-level changes in different men evaluated in different years, not within-individual changes. Cohort effects (differences between birth cohorts) versus period effects (impacts affecting all men at a given time) versus age effects cannot be fully disentangled. DNA integrity testing incompleteness: DNA fragmentation and denaturation testing was not performed on all samples (performed on approximately 45% of total cohort), and the subset tested may have been clinically selected (e.g., men with very poor conventional parameters, recurrent pregnancy loss, or specific clinical indications), introducing potential selection bias in DNA integrity analyses. Lack of hormonal data: serum hormone levels (follicle stimulating hormone (FSH), luteinizing hormone (LH), testosterone, and inhibin B) were not systematically available for this cohort, limiting our ability to distinguish primary testicular failure from secondary hypogonadism or to correlate endocrine profiles with semen quality trends. Morphology assessment methodology: while morphology was assessed using the Kruger strict criteria throughout the study, this parameter is inherently subjective and has higher inter-observer variability than concentration or motility. Although our embryologists underwent regular training and quality control, we cannot entirely exclude that some of the observed decline could reflect subtle changes in scoring stringency over the 12-year period spanning 2013–2024. However, the magnitude of decline (from 12.1 to 5.0%), its consistency across multiple assessors, and its concordance with changes in other parameters argue that it primarily reflects true biological deterioration. Laboratory transitions between WHO editions: the transition from WHO 5th edition (used in 2019–2020) to 6th edition (2021–2024) introduced some methodological refinements, although both editions use fundamentally similar assessment principles. Any biases introduced by this transition would affect 2021–2024 uniformly and cannot explain the continuous year-over-year declines observed within each WHO edition period. Ecological fallacy: when discussing potential environmental exposures or lifestyle factors, we are making population-level inferences without individual-level exposure data. Associations observed at the population level may not hold at the individual level.

Despite these limitations, the consistency, magnitude, and regional breadth of the observed declines, combined with their concordance with global trends and biological plausibility, support the conclusion that semen quality deterioration is a real and substantial public health concern in North Africa.

Future prospective studies should investigate the mechanistic pathways by which COVID-19 and environmental exposures impair spermatogenesis (Santi *et al.* 2017, Agarwal *et al.* 2021). In addition, regional public health initiatives should address modifiable risk factors for male infertility, particularly in younger populations (Skakkebaek *et al.* 2016, Okonofua *et al.* 2022).

The findings of this study have important clinical and public health implications for North Africa. From a clinical perspective, the increasing prevalence of oligoasthenoteratozoospermia (OAT) and severe teratozoospermia means that a growing proportion of infertile couples in this region will require assisted reproductive technologies (ARTs), specifically intracytoplasmic sperm injection (ICSI), to achieve pregnancy. This has economic implications, as ARTs are expensive and not universally covered by insurance in North African countries, potentially exacerbating health inequities. Healthcare systems in the region need to scale up ART infrastructure, training, and accessibility to meet this growing demand.

From a public health perspective, the data suggest that male fertility decline in North Africa may be reaching critical levels that could affect population demographics and family formation. While current total fertility rates remain above the replacement level in most North African countries (approximately 2.3–3.0 children per woman as of 2022), male fertility impairment could contribute to declining birth rates observed in recent years, increase the time to pregnancy at the population level, and create substantial psychosocial and economic burdens for affected couples.

Preventive strategies should focus on modifiable risk factors by reducing exposure to environmental toxicants through regulation of EDCs in consumer products, enforcement of air quality standards, and promotion of organic agriculture with reduced pesticide use. Public health campaigns targeting lifestyle factors, including obesity prevention, smoking cessation, promotion of physical activity, and nutritional optimization, are warranted. Occupational health interventions to minimize workplace exposures to heat, radiation, heavy metals, and gonadotoxic chemicals must be implemented. Surveillance systems for male reproductive health should be established to monitor trends and identify emerging risk factors. Male reproductive health should be incorporated into pre-conception care guidelines and general health screening for men of reproductive age. Given the potential long latency between exposure and reproductive outcomes (many reproductive toxicants act during fetal development, puberty, or early adulthood), interventions implemented today may not show measurable benefits for years or decades. This not only makes early action imperative but also challenges political will and resource allocation.

Future studies should prioritize i) biomonitoring of environmental contaminants correlated with semen parameters, ii) prospective cohorts with detailed exposure assessment enabling causal inference, iii) general population studies to distinguish infertility-specific versus population-wide trends, iv) COVID-19-specific longitudinal studies with documented infection status and serial analyses, v) intervention trials targeting lifestyle factors and environmental exposures, and vi) fertility outcomes research linking semen parameters to pregnancy and live birth rates in North African populations.

## Conclusion

This large retrospective multicentric study of 21,585 men from three North African countries provides compelling evidence of continued and accelerating deterioration in semen quality between 2019 and 2024. Compared to a historical cohort from the same laboratories (2013–2018), we observed alarming declines of 27.6% in sperm concentration, 20.5% in progressive motility, and 58.7% in normal morphology. The proportion of men presenting with normozoospermia decreased from 32% to less than 19%, while severe oligoasthenoteratozoospermia increased from 16 to 28%.

Temporal analysis revealed complex patterns suggesting multiple etiologic factors. A sharp decline in concentration and motility in 2020, coinciding with the COVID-19 pandemic, was followed by partial but incomplete recovery, with 2024 values remaining below the 2019 baseline. In contrast, sperm morphology demonstrated continuous linear deterioration throughout the entire 6-year period with no inflection point or recovery, suggesting chronic cumulative environmental exposures independent of pandemic-related acute effects.

Significant inter-country variation was observed, with Libyan men showing the best semen parameters and Algerian men the poorest, likely reflecting differences in environmental exposures, healthcare infrastructure, infection burden (evidenced by higher leukospermia rates in Algeria), and socioeconomic factors. However, declining trends were consistent across all countries, indicating region-wide determinants.

These findings position North Africa among the regions experiencing the most severe declines in male reproductive health globally and raise concerns about potential demographic and public health consequences. The multifactorial etiology likely includes environmental contamination with EDCs, air pollution, pesticide exposure, adverse lifestyle factors (obesity and smoking), infectious/inflammatory processes, and possibly lasting or recurring impacts of the COVID-19 pandemic.

Urgent action is needed at multiple levels: i) implementation of environmental regulations to reduce exposure to reproductive toxicants, ii) public health campaigns targeting modifiable lifestyle risk factors, iii) establishment of surveillance systems for ongoing monitoring of male reproductive health, iv) expansion of accessible, affordable fertility care services, and v) investment in mechanistic research to identify specific etiologic agents and develop targeted interventions. Without such measures, male fertility decline in North Africa is likely to accelerate further, with profound implications for individual couples, healthcare systems, and society.

## Declaration of interest

The authors declare that there is no conflict of interest that could be perceived as prejudicing the impartiality of the work reported.

## Funding

This research did not receive any specific grant from any funding agency in the public, commercial, or not-for-profit sector.

## Author contribution statement

HB, MB1, and MB2 conceived the study; MB1 and WZ designed the study; WZ handled the software; MB1, MB2, and HB validated the results; WZ performed formal analysis and curated the data; MB1 was involved in the investigation; HB arranged resources, administered the project, and acquired funding; MB1 drafted the original manuscript; ML and MB1 reviewed and edited the manuscript; HB and MB2 supervised the study. All authors read and agreed to the published version of the manuscript.
